# Does team-based learning improve performance in an infectious diseases course in a preclinical curriculum?

**DOI:** 10.5116/ijme.5895.0eea

**Published:** 2017-02-08

**Authors:** Kathryn C. Behling, Rose Kim, Matthew Gentile, Osvaldo Lopez

**Affiliations:** 1 Department of Biomedical Sciences, Cooper Medical School of Rowan University, Camden, NJ, USA; 2 Department of Medicine, Division of Infectious Diseases, Cooper University Hospital, Camden, NJ, USA; 3 Office of Medical Education, Cooper Medical School of Rowan University, Camden, NJ, USA

**Keywords:** Learning improvement, medical students, learning perception, team-based learning

## Abstract

**Objectives:**

To examine whether introduction of Team-based Learning (TBL) improves student learning resulting in improved performance on final examination questions and decreased failures in an infectious diseases course.

**Methods:**

To improve mastery of course content, we designed an intervention, which provided weekly TBL exercises in study years 2 and 3 to review concepts presented during didactic lectures and laboratory exercises.  The remaining course structure and content was essentially unchanged. All students taking the course (n=50 in year 1, n=64 in year 2, and n=72 in year 3) participated in this study. Student final examination performance and performance on individual final examination questions were collected and analyzed for changes in response to the study intervention.

**Results:**

Addition of weekly TBL exercises improved student performance on the course final examination as demonstrated by a statistically significant increase in the distribution of correct answer percentages for questions in common between the final examinations in years 1 and 2 and between years 1 and 3 (t_(99)_ = 3.1454, p<0.05 and t_(99)_ = 4.1268, p<0.01, respectively; Student-Newman-Keuls).  There was no statistical difference (t_(97)_ = 0.9814, p> 0.05; Student-Newman-Keuls) in the distribution of correct answer percentages between years two and three. There was also a decrease in final examination failures in years two and three.

**Conclusions:**

The results suggest that TBL could be used to improve mastery and retention of course content in a preclinical infectious diseases course. Weekly exercises allow students to identify and ameliorate weaknesses in understanding and make adjustments early in the course.

## Introduction

In our fast paced, ever changing world of medicine, it is essential for physicians to access, analyze, and apply new medical knowledge that is needed for up to date, evidence-based, patient care. As such, instructional strategies in medical schools must foster an educational environment that promotes the ability of future physicians to perform their own learning needs assessments, design their own learning objectives, and access the resources necessary to meet these learning objectives.  Active learning methods can help students achieve these goals as well as improve student outcomes by promoting critical thinking, deep learning, and higher-learning skills.^1^ These methods also encourage students to take personal responsibility for their learning, giving them a sense of autonomy.  Research has shown that when students feel that they are in control of their learning, they become intrinsically motivated, which results in improved understanding.^2^ Additionally, practice in taking personal responsibility for learning provides future physicians with the skills necessary for life-long learning.

TBL is a structured instructional strategy that encourages peer-to-peer teaching to reinforce and expand on material learned by individual students independently.^3^ Educational content is provided in the form of assigned lectures, readings and/or videos, which students complete prior to the TBL exercise.^4^ The TBL technique was first introduced in the 1970’s  by Larry Michaelsen at the University of Oklahoma. Michaelsen was concerned that with increasing class size, he was not able to monitor whether the students were thinking critically about the presented lecture material.^4^ Since then, TBL has been used in a variety of educational environments to promote critical thinking and active learning.  As such, the use of TBL has expanded to many areas of higher education including nursing,^5^ medical,^6^^-^^8^ pharmacy,^9^ law,^10^ veterinary,^11^ and dental schools,^12^ and medical residency,^13^ among other disciplines.

In the first iteration of the infectious diseases (ID) course at our institution, students struggled to master and retain course content, as evidenced by a 16% student failure rate on the final examination.  On review of the literature, we identified TBL as a pedagogical technique that had the potential to improve student performance. Notably, in a review of the literature regarding the use of TBL in medical education, Fatmi et al found that seven of ten studies showed a statistically significant increase in student knowledge scores.^14^ Additionally, another study reported that the use of TBL also improved success of students who were at risk for course failure.^6^ Although we found ample literature showing the benefit of TBL in medical education, we were unable to identify any studies which addressed the use of TBL in an infectious diseases preclinical medical school course.

Given the documented success of TBL in medical education, we designed a pilot program using TBL to improve student learning outcomes in the ID course offered as part of the basic science curriculum at our medical school.  Through this program, we introduced weekly TBL exercises, which were intended to reinforce material previously covered in lectures and laboratory sessions.  As such, this study was designed to test our hypothesis that TBL exercises would improve student learning of course content resulting in improved performance on the course final examination.

## Methods

### Research design

We used a cohort study design to examine the effect of TBL on student performance in the Infectious Diseases (ID) course at our institution.  Three different student cohorts were compared.  The first cohort included fifty students who took the ID course during the first year of the study when TBL exercises were not used.  The second cohort included sixty-four students who took the ID course in the second year of the study after introduction of three weekly TBLs at the beginning of weeks 2, 3, and 4 of the course.  The third cohort included seventy-two students who took the ID course in the third year of the study after introduction of four weekly TBLs at the beginning of weeks 1, 2, 3, and 4 of the course. Lectures and case-based learning activities did not change during the three years of the study.

### Study population

All studies were conducted with approval of the Institutional Review Board of Rowan University. Cooper Medical School of Rowan University (CMSRU) is a Liaison Committee on Medical Education (LCME) accredited, medical degree granting, allopathic medical school in the US.   In the three years of the study, all students taking the ID course were enrolled in this study (year 1: n=50, year 2: n=64, year 3: n=72). Student examination data was de-identified prior to data analysis in accordance with the IRB protocol.

### Study setting

The ID course is a four-week course in the second semester of the first-year, basic science medical school curriculum at CMSRU. This course reviews the fundamental basis of identification, diagnosis, and management of infectious diseases. 

### Team-based learning exercises

TBL exercises were conducted as described by Michaelsen and Sweet.^3^ Details regarding TBL team formation and student assessment are reported here using the guidelines for reporting TBLs in the medical and health sciences education literature.^15^ Briefly, student teams were composed of six students.  Students did not assist in determination of team membership. When composing these teams, we considered diversity in sex, previous education, ethnicity, and national background.

Each TBL exercise accounted for 3% of the student’s overall course grade.  The distribution of assigned points for the components of the TBL exercises (IRAT/GRAT) was determined solely by the faculty without input from the students. In the second year, TBL exercises were graded as follows: IRAT score 33.33 % and GRAT score 66.67%. In year three, the grading structure was changed to the following: IRAT score 30%, GRAT score 60% and application score 10%.  No student peer-review process was used, and therefore, student opinion of classmate performance was not included in student grading.

TBL was not used in year 1 of the study.  In year 2 of the study, TBL exercises were held on the first day of weeks 2, 3 and 4 of the ID course.  These exercises were designed to foster mastery and application of concepts learned in the didactic lectures and laboratory sessions from the previous week. In year 3, a new TBL exercise was added, which took place on the first day of the ID course. This exercise was designed to review basic microbiology and immunology topics covered in the first semester of the first year curriculum.

### Student examination data

The ID summative final examinations contained approximately 100 multiple-choice, single best answer questions.  There were fifty-three questions in common between the summative final examinations offered in years one through three, and the percentage of students correctly answering each question (p-values) was collected and de-identified for the three years.

Data regarding performance on the ID final examinations in years one through three, including number of failures (examination scores less than 70%), were also collected.  To control for variations in the student cohort from year to year, Hematology/Oncology (Heme/Onc) data regarding final examination performance, including number of failures, were also obtained.  TBL was not used in the Heme/Onc course.

### Student feedback data

In year two, anonymous student feedback was obtained using paper surveys distributed to the students at the end of each TBL session. Question asked included: 1) The iRAT questions reflect the didactic material presented on the included subject matter.  2) The gRAT helped me to better understand the subject matter.  3) The application questions helped me to acquire a deeper understanding of the subject matter.  Students were asked to rate survey statements using a Likert scale where 1 = strongly disagree, 2 = somewhat disagree, 3 = neutral, 4 = somewhat agree, 5 = strongly agree.

In year three, anonymous student feedback was obtained through the CMSRU faculty evaluation system. In this system, feedback was only requested of approximately 50% of the students taking the course.  Questions asked included: 1) The objectives of the session were clear. 2) The session was well organized. 3) The session was relevant to my education. 4) The content helped me meet session objectives. 5) The session content was related to course objectives. 6) The session stimulated me to want to learn more about the subject. 7) The faculty maintained my interest. 8) The faculty demonstrated appropriate knowledge.  9) The faculty explained the material clearly. 10) The faculty used questions and student participation effectively. 11) The faculty demonstrated professionalism. Students were asked to rate these statements using the Likert scale described above. The session evaluation form also included free response questions, including “Comments about today’s session”.  Responses to this question were also collected.

### Data analysis

Student performance data from the three academic years were compared. Specifically, the p-values of the fifty-three questions in common between the three exams were compared using a Kruskal-Wallis one way analysis of variance of ranks followed by a pairwise multiple comparison (Student-Newman-Keuls) using IBM SPSS Statistics for Windows, version 22 (IBM Corp., Armonk, NY, USA). Final examination scores for the ID and Heme/Onc courses were determined by combining scores from the practical and written examinations proportional to the distribution of educational content assessed. Final examination score means and standard deviations were calculated.

Student feedback data was analyzed to calculate means and standard deviations of scores. For the student evaluations from year two, student evaluation scores were averaged and standard deviations were calculated. For the student evaluations from year three, scores from the first eleven questions were averaged, and this number was used to represent the overall evaluation score for the session. The student evaluations from year three also included a free response question, “Comments about today’s session”. Two of the authors (KB and OL) independently scored the responses to this question as positive, neutral, or negative, and their consensus determinations are shown in Table 1. Responses including “N/A” and “.” were not included in the analysis.

**Table 1:** Student evaluation of Team-Based Learning sessions in year three

TBL	Overall Session Score Mean (SD)	Comments about today’s session			
		Positive	Neutral	Negative	Not answered
TBL 1 (n=35)	4.0 (0.74)	14	4	3	14
TBL 2 (n=35)	4.0 (0.91)	8	8	4	15
TBL 3 (n=33)	4.1 (0.82)	7	7	2	17
TBL 4 (n=34)	4.2 (0.76)	12	2	1	19

## Results

### Individual question performance

**Figure 1:**
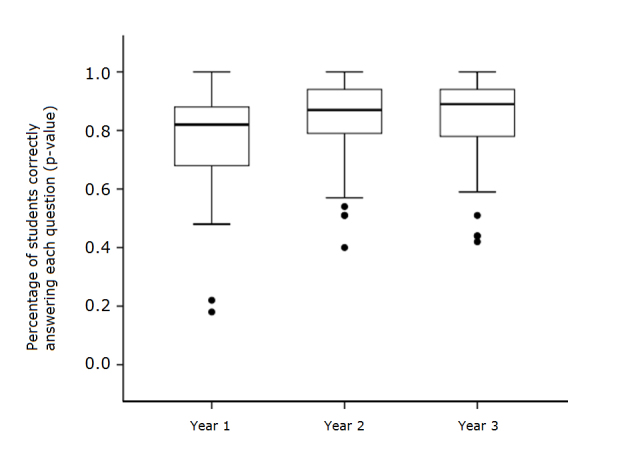
Performance on individual ID final examination questions

There were fifty-three questions in common between the year one, year two, and year three Infectious Diseases (ID) final examinations.  The distributions of the percentages of students correctly answering each question (p-value) in year one (n= 50 students), year two (n = 64 students), and year three (n = 72 students), were compared using a Kruskal-Wallis one way analysis of variance (ANOVA) of ranks followed by a pairwise multiple comparison (Student-Newman-Keuls). A Kruskal-Wallis one-way ANOVA revealed a significant difference in the three experimental groups (χ^2^_(__2)_=11.2972, p=0.0035). Figure 1 shows the performance on individual final examination questions. Pairwise multiple comparisons (Student-Newman-Keuls) demonstrated a significant difference between the year one and year two examinations (t_(__99)_ = 3.1454, p<0.05), and between the year one and year three examinations (t_(99)_ = 4.1268, p<0.01). However, there were no significant differences between the year two and year three examinations (t_(__97)_ = 0.9814, p> 0.05).  Thus, we were able to show a statistically significant improvement in the distribution of p-values on fifty-three questions in common between the final examinations offered in years one, two and three after addition of TBL in years two and three (Figure 1). The major difference between the year one, year two, and year three offerings of the ID course was the addition of TBL exercises in years two and three. The remaining structure of the course, including lectures, laboratory sessions, and case-based learning curricular content, remained essentially unchanged. The results suggest that the addition of TBL exercises improved student performance on final examination questions.

### Final examination performance

Final examination performance in the ID course improved from year one (M= 79.51, SD= 8.67) to year two (M= 84.02, SD= 8.94) and decreased slightly in year three (M=82.68, SD=9.01).  However, the percentage of failures on the final examination decreased from 16% in year one, to 6.3% in year two and 2.8% in year three. To control for differences in the student cohort from one academic year to the next, we chose to look at performance in another second semester, first year course, the Heme/Onc course.  This course does not use TBL, and there were no significant changes in course content and presentation over the three years of the study.  We found that over the same three-year period, final examination grades in the Heme/Onc course increased from year 1 (M= 83.95, SD= 7.58) to year 2 (M= 86.54, SD= 7.45) and to year 3 (M= 88.12, SD= 6.9).  However, there was little change in the percentage of final examination failures with a 2.0% failure rate in year one, 4.7% failure rate in year two, and 1.4% failure rate in year three.  Our data suggest that addition of TBL exercises to the ID course improved performance on the final examination and, in particular, that of the lower performing students that are most at risk for failure. 

### Student perception

Students completed anonymous surveys about the TBL exercises in years two and three of the study.  These evaluations showed that most students had positive perceptions regarding the TBL exercises.  In year two, most students felt that the iRAT reflected the didactic material (student evaluation score averages - TBL 1: 4.5 (SD= 0.68), TBL 2: 4.6 (SD=0.62) and TBL 3: 4.8 (SD= 0.52) out of 5), and the gRAT sessions helped them to understand course content (student evaluation score averages - TBL 1: 4.6 (SD= 0.67), TBL 2: 4.5 (SD= 0.80) and TBL 3: 4.7 (SD= 0.70) out of 5).  Most students also felt that the application questions helped them acquire deeper understanding of course content (student evaluation score averages – TBL 1: 4.0 (SD= 1.0) and TBL 3 4.1 (SD= 1.1) out of 5), there was no application in TBL 2. In year three, average evaluation scores for each of the four sessions ranged from 4.0 to 4.2 out of 5 (Table 1).  Many of the student comments regarding the sessions were also positive (Table 1) with students commenting on the utility of TBL for keeping up with and applying course content.

## Discussion

The addition of TBL exercises to the ID course in years two and three of our study resulted in improvement in student final examination performance (Figure 1).  We were able to show a statistically significant improvement in the distribution of p-values on fifty-three questions in common between the final examinations offered in years one, two and three after addition of TBL in years two and three (Figure 1).  We also saw a decrease in final examination failures in years two and three as compared to year one.  Final examination performance in the Heme/Onc course served as a control for changes in the student cohort over the course of the study, and this course did not show significant differences in final examination grades or failures.  In addition to helping students perform better on the ID final examinations, students found the TBL exercises enjoyable and rated them highly on student evaluations.

We believe that TBL exercises improved student performance because they encouraged students to keep pace with course content and gave students opportunities to identify and dispel misconceptions about course concepts through peer-to-peer teaching. Indeed, on the session evaluation forms, students stated that TBL helped them to stay on track with and practice application of course concepts. Additionally, we feel that the decrease in the number of final examination failures is consistent with TBL assisting poorer performing students in the class.  This finding is consistent with prior research that suggests that TBL significantly helps struggling students.^6^

In our interactions with the students throughout the study, we noticed that students had difficulty with mastery of ID course concepts.  We believe this was due in part to the timing of the ID course within our institution’s basic science curriculum as it is the first “organ system” course in the first year curriculum.  Prior to this course, students are taught primarily basic science content in a more teacher-centered approach.  In ID, they are forced to transition to the discussion of clinical cases in a more student-centered, self-directed learning approach.  The TBL sessions assist in this transition by encouraging students to review course content independently prior to the sessions as well as thinking critically about course material during the sessions through both gRAT and application exercises. Other researchers have also found TBL to be an effective strategy to strengthen critical thinking.^16^

Anonymous student feedback obtained in years two and three demonstrated positive student perceptions of the TBL sessions.  On review of the literature, Gray et al also reported similar positive student feedback on use of TBL in Zimbabwe for a competency-based HIV curriculum for final-year students.^17^ Nevertheless, in our study, there was a small decline in the year three evaluations as compared to the year two evaluations.  This is in apparent contradiction to other studies, which have demonstrated improved student satisfaction with establishment of the TBL methodology over time.^18^ We feel that the change in evaluation scores at our institution might be due to a change in grading structure for the TBL exercises.  In our study, the application exercises were ungraded in year two and graded in year three.  In year three, we received feedback from the students that grading of the application exercises increased student anxiety.  Medical students tend to be very conscious about their grades, and losing even a very small percentage of a grade may increase anxiety in some students.^19^ Therefore, the lower evaluation score may reflect the distress caused by increased assessment. In one extensive review of studies examining use of TBL in health professions education in Europe, the Middle East, and the US, Fatmi et al found that TBL generally improves student performance but can have mixed student satisfaction.^14^ Ultimately, each institution must determine how they plan to grade TBL exercises, balancing the impact of assessment on increasing student anxiety with providing motivation to adequately prepare for the exercises.

### Limitations of this study

Our study conclusions are limited by numerous factors inherent to research on the effects of novel teaching methods on educational outcomes in medical schools.  The cohort of students in each year was different, which may have introduced variability including, but not limited to, differences in academic metrics prior to matriculation such as MCAT scores and grade point averages, educational background such as prior experience with microbiology and immunology, and prior experience with small group educational sessions.  Because we could not control for these factors, we chose to perform a semi-quantitative comparison to another first year, “organ system-based” course, Heme/Onc, which is a five-week course that takes place after ID.  In the Heme/Onc course, we found that over the study period, students performed similarly, especially with regard to failure rates, while failure rates improved in the ID course in years two and three after the addition of TBL.  These findings suggest a positive effect of TBL in the ID course. 

## Conclusions

In summary, in agreement with our hypothesis, the introduction of weekly TBLs facilitated learning of course content resulting in improved performance on the course final examination. Additionally, our students found TBLs helpful in keeping up with and improving understanding of course content. We think that this technique has the potential for use in other areas of the pre-clinical curriculum as well as in clinical undergraduate and graduate medical education curricula.

### Acknowledgements

We would like to thank Dr. Michael O’Leary, Associate Professor and Dr. Gonzalo Carrasco, Assistant Professor, Dept. of Biomedical Sciences, Cooper Medical School of Rowan University (CMSRU) for their help with statistical analysis, Ms. Susan Cavanaugh, MPH, EBM Librarian I, CMSRU-Cooper University Hospital for her help with reference citations, and Dr. William Kocher, Chair, Dept. of Biomedical Sciences, CMSRU who kindly shared his data from the Hematology-Oncology Course for use in this study.

### Conflict of Interest

The authors declare that they have no conflict of interest.
